# The Efficiency of Kinesiotherapy versus Physical Modalities on Pain and Other Common Complaints in Fibromyalgia

**DOI:** 10.3390/life14050604

**Published:** 2024-05-08

**Authors:** Daniela Matei, Rodica Trăistaru, Vlad Pădureanu, Taina Elena Avramescu, Daniela Neagoe, Amelia Genunche, Anca Amzolini

**Affiliations:** 1 Department of Medical Rehabilitation, University of Medicine and Pharmacy Craiova, 200349 Craiova, Romania; mateidana30@yahoo.com (D.M.); rodicatraistru@gmail.com (R.T.); 2 Department of Internal Medicine, University of Medicine and Pharmacy Craiova, 200349 Craiova, Romania; daniela.neagoe@umfcv.ro (D.N.); amzolinianca@yahoo.com (A.A.); 3 Sport Medicine and Physiotherapy, University of Craiova, 200585 Craiova, Romania; taina_mistico@yahoo.com

**Keywords:** kinesiotherapy, physical modalities, pain, fibromyalgia

## Abstract

Due to its variety of signs and symptoms, there have been numerous attempts to treat fibromyalgia (FM), but a cure has yet to be established. The aim of this study was to evaluate the effects of a complex kinetic therapy program and a combined physical modality program on pain and other common symptoms of FM. Patients and methods: A total of 78 female patients were included in this study; 39 subjects underwent a kinesiotherapy (KT) intervention (combining aerobic and Pilates exercises), and 39 participated in a physical modality (PM) program (including electrotherapy (TENS and low-laser therapy) and thermotherapy). Results: Regarding the parameter of pain assessment, kinesiotherapy demonstrated its superiority both during the treatment period and in the evaluation 3 months after therapy cessation. Both in terms of patient-reported pain (inter-group comparisons: *p* = 0.000 at T3) and the examination of tender points (inter-group comparisons: *p* = 0.000 at T3), as well as the algometric assessment, pain was alleviated by the two forms of applied kinetic therapy. The observed functional impairment was statistically significantly influenced (*p* = 0.001) at the end of the kinetic program application, while for the perceived functional impairment, neither therapy proved superiority over the other at any point of evaluation (inter-group comparisons: *p* = 0.715 at T3). Regarding the influence of the emotional consequences implied by fibromyalgia, neither the forms of kinesiotherapy nor the chosen physical modalities proved superiority at any point of evaluation (HAQ anxiety inter-group comparisons: *p* = 0.000 at T3). In conclusion, even though kinesiotherapy had superior influences on fibromyalgia pain in the studied group, the current research lends credence to the significance of non-pharmacological therapy in managing fibromyalgia. Participants demonstrated positive advancements in subjective and objective pain assessments, as well as improvements in functional and emotional well-being.

## 1. Introduction

Fibromyalgia (FM) is characterized by persistent, widespread, non-inflammatory musculoskeletal pain accompanied by chronic fatigue and various clinical and emotional symptoms [1], without any evident underlying pathology. The women/men ratio varies from 7:1 [2] to 10:1 [3], with the peak onset being considered between 30 and 50 years old and the 5th decade of life being the most affected [4]. The prevalence of FMS in the adult population of the United States is estimated at 2.0% [5], whereas in Europe, the incidence varies significantly, ranging from 0.7% in Denmark [6] to 5.5% in Italy [7]. Although the exact cause of FM remains unknown, it is believed that a combination of biological, psychological, and social factors contributes to pain amplification and central sensitization to peripheral stimuli [8]. Trigger factors for FM may include viral or bacterial infections, acute illness, physical injury, surgery, or stressful psychosocial situations [9].

Similar to other rheumatic disorders [10,11,12,13,14], fibromyalgia leads to disability and imposes substantial healthcare costs and productivity loss [15]. Apart from the diagnostic standards outlined by the American College of Rheumatology (ACR) in 2010 [16], supplementary criteria involve various symptoms linked to widespread pain, including cognitive issues, depression, sleep disturbances, and numerous somatic symptoms impacting daily functioning [15].

Research has shown a connection between two aspects of this condition: pain and muscle endurance [17]. Numerous studies have indicated a negative relationship between pain, pain catastrophizing, and physical ability among individuals with FM [18,19]. Regarding cardiovascular function, there were no notable variances observed in heart rate; however, the maximum oxygen consumption (ranging from 23 to 30 mL/kg min) was lower compared to the range (30–40 mL/kg min) typically seen in healthy individuals who are not trained [20]. The lack of organic neuromuscular changes suggests that physical deconditioning in FM patients follows a sedentary lifestyle pattern [21].

Different treatments, such as medication, psychological interventions, physical modalities, and exercise therapy, have been explored as potential remedies for FM [22].

Kinesiotherapy, which involves the use of different types of structured exercises performed in a repetitive and adapted manner, is a form of physical activity with the primary goal of preserving or improving the patient’s functional status [23]. Therefore, the most common effects of training include the reduction in stress, depression, and anxiety levels, partly due to the psychological benefits of physical activity and partly due to the sense of community with others who have similar complaints and problems [24]. Certain studies have provided insufficient evidence regarding the long-term effects of kinesiotherapy [25]. Concerning exercise intensity, most experts recommend a gradual progression starting with low-intensity exercise [26,27], following the “start low and go slow” principle to eventually achieve at least moderate intensity [28]. Strength training programs should begin with resistance levels lower than the age-predicted norms [26]. If significant pain or fatigue arises, exercise session intensity and duration should be reduced [29], and intensity can be increased by 10% after two weeks if symptoms are not aggravated [26]. Training frequency varies from one to six sessions per week, with three times a week being the most common [30]. The duration of exercise ranges from five to fifty minutes per session, spanning from four [31] to thirty-two weeks [32], with an average duration of approximately twelve weeks.

In addition to kinesiotherapy, physical modalities have been explored to improve FM symptoms. While kinetic therapy has shown favorable effects, the evidence supporting the use of physical modalities is much weaker [33]. Although transcutaneous electrical neuromuscular stimulation (TENS) works by reducing central excitability and activating central inhibition pathways, there is little evidence regarding its efficacy in FM patients [34]. Thermotherapy procedures, such as local application of heat or cold, have demonstrated good results in controlling pain and reducing the number of tender points [35,36].

Massage is another therapeutic strategy frequently used to manage the most important complaints in patients with FM [37].

Other therapeutic options for FM include balneotherapy, which involves water-based treatments, and thalassotherapy, which uses marine products. However, there is currently only low-to-moderate evidence supporting the effectiveness of balneotherapy in treating FM [38].

The limited comprehension of the causes and pathogenic processes underlying FM leads to considerable direct and indirect healthcare expenses [39]. Certain experts propose that a multidisciplinary strategy, integrating different forms of movement therapy or physical methods with other established effective treatments within a carefully designed program, may offer the most hopeful approach for managing FM [30,40].

It is well known that the adherence of fibromyalgia patients to treatment is reduced, on the one hand, due to fluctuations in symptomatology [41] and, on the other hand, due to the absence of a concrete therapy for this condition. Currently, there is not sufficient support in the literature for the use of multicomponent non-pharmacological therapies for FM patients. This research aims to present the rationale and methods of a randomized study aimed at evaluating the efficacy of treatments involving kinesitherapy or the combination of physical modalities in influencing pain, improving function, and enhancing quality of life in patients with known FM.

## 2. Materials and Methods

### 2.1. Design Overview

In this randomized, longitudinal, and non-inferiority study, 78 female patients previously diagnosed with fibromyalgia (FM) were randomized to 12 weeks of either kinesiotherapy (*n* = 39) or physical modalities (*n* = 39), and the treatment period was then evaluated. In order to evaluate not only the impact on pain but also the subjective and objective changes in terms of functional status and quality of life resulting from both methods in the short term, we conducted a new assessment at 3 months post-treatment.

### 2.2. Study Group

All the patients were gathered from the Rehabilitation and Physical Medicine Departments of the County Emergency Hospital and the Filantropia Municipal Hospital of Craiova, Romania. Initially, 39 patients agreed to participate in the KT sample, but ultimately only 34 participants were included in the database for attending at least 70% of the program sessions, resulting in an 87.17% participation rate among the initially interested patients. Similarly, 39 patients were initially included in the PM sample; 30 of them were ultimately considered for the analysis due to attending at least 70% of the program sessions, resulting in 76.9% of the initial patients completing the program.

Sample size: Using the most common values for the level of confidence and the power of the test, confidence of 95% (α = 0.05) and power of 80% (β = 0.2), and considering that the average effect size (ES—the ratio between the score difference and the pooled standard deviation) to be observed in the analyzed variables is 0.5, we can use the following formula to approximate the required sample size: *n* = Z2/ES2, where Z = z1-α + z1-β. For our chosen values, each of the two sample groups should have a minimum of 30 patients with all recorded data.

The inclusion criteria were as follows: (1) being over 18 years of age, (2) signing an informed consent form to participate, (3) having a confirmed previous diagnosis of FM (according to the 2010 ACR criteria), (4) speaking and understanding the language perfectly, (5) lack of prior exposure to TENS, low-level laser therapy, massage, or kinesiotherapy (aerobic and/or Pilates exercises), and (6) constant analgesic treatment (stable doses) for at least 1 month prior to study inclusion and constant maintenance of analgesic treatment throughout the entire treatment period applied. The general exclusion criteria included the following: (1) illiteracy, (2) alcoholism, (3) severe psychiatric disorders, (4) uncooperative patients, (5) pregnancy, (6) traumatic injuries, and (7) associated conditions that contraindicate physiotherapy (such as hematological diseases, pacemakers, tuberculosis, malignant tumors, and so on).

According to the predetermined inclusion and exclusion criteria, eligible patients were informed about the study and provided with the appropriate informed consent. During the patient recruitment period, all consecutive patients with fibromyalgia who met the inclusion/exclusion criteria were only females.

They were then scheduled for an appointment at the hospital, where they underwent an interview and a battery of assessments, including pressure pain sensitivity measurement (algometry) and tender point examination (T1), conducted by a specialized physiotherapist. Subsequently, the subjects were required to attend 36 interventional sessions, and they were reevaluated using the same procedures at the end of the program (T2). A follow-up assessment was also conducted 3 months later (T3).

### 2.3. Types of Applied Treatments

Kinesiotherapy group: The patients included in KT were divided into groups of a maximum of 10 patients and attended three sessions of kinesiotherapy per week. Each session included the following types of exercises: aerobic and Pilates exercises. A rehabilitation physician and at least one kinetic therapy specialist conducted and supervised the session, which lasted 20 min at first, slowly increasing in duration and intensity as weeks went by, reaching a maximum duration of 1 hour towards the end of the study. The goals of the kinetic program were for the patients to manage the following: knowing how physical activity can influence FM symptoms; understanding that regular exercise can improve physical function, pain tolerance, and mood; experimenting with how moderate physical activity can lead to adopting correct postures and, by regularly practicing it, generalizing these postures in the activities of daily living (ADLs); improving muscle tone; enhancing the safety and commodity of ADLs; increasing the force and flexibility of the muscular system in order to improve one’s physical capacity; improving mobility and balance; and helping achieve relaxation. All patients received a layout of each session with suggestive images in order to enable them to perform the exact same exercises at home. Each session included warm-up exercises as well as exercises for the upper and lower limbs, and the exercises were presented with two alternatives, depending on the doctor’s and patient’s decision. The goal was to enable every patient to achieve the same result without forcing them to go over the limit. Moreover, in order to improve relaxation, the exercises were performed to a soft music soundtrack. Kinesiotherapy, including both aerobic exercise and regular Pilates sessions, was employed with the aim of enhancing pain perception and improving functional status [42,43].

Physical modality group: The patients from the second group attended 36 sessions of physical modalities, three sessions per week. Each session included two forms of electrotherapy (TENS and low-level laser) and thermotherapy:

- The TENS device (BTL 4000, Astar Polonia, Bielsko Biala, Polond) used in this study allowed for the establishment of the following parameters for all patients: TENS 200 μs, 2 and 100 Hz, 60 mA, 20 min; electrodes were positioned on painful areas, and the intensity was incrementally raised to a level that was strong but still manageable.
- The low-level laser (LLL) device (BTL 4110, Astar Polonia, Bielsko Biala, Polond) allowed the application of therapy with the following parameters: 2 J/cm^2^, 3 min each tender point.
- Thermotherapy involved applying a mudpack at a temperature of 40–45 °C for 20 min to areas experiencing pain. Subsequently, the body was wrapped in both insulating and dry linen sheets. Following the removal of the mud, a 10 min dry body pack was utilized to enhance the overall effectiveness of the procedure.

The procedures were combined in such a manner for each patient that they would cover at least one part of every area of the body: the spine (cervical, thoracic, lumbar, and sacral), the upper limbs (shoulders, arms, elbows, forearms, and hands), and the lower limbs (hips, thighs, knees, calves, ankles, and feet). The objective of the physical modality intervention was to improve the patient’s condition by combining an analgesic effect, both local and at a distance, by activating the reflex areas (TENS) [44]; a muscle relaxant effect and an indirect effect of reducing local pain through vasodilation and accelerating metabolism (thermotherapy) [45]; a biotrophic effect with the corresponding circulatory activation and local pain reduction (LLL) [46]; a sedative effect with psychological benefits (thermotherapy); and an improvement in relaxation and well-being (all the mentioned physical modalities).

### 2.4. Ethical Considerations

The patients included in the present study signed an information and acceptance form, i.e., an informed consent, to be included in the present study. The study protocol was approved by the local Ethics Committee (no. 80/13.05.2022) and was conducted following the Helsinki Declaration.

### 2.5. Parameters and Instruments

Assessment of different types of parameters is very important when conducting kinesiotherapy and physical modalities, regardless of the disease [47]; hence, we attempted to evaluate the most important parameters in FM.

Pain: For this parameter, we assessed the patients across three measures:

(a) Self-reported pain (11-point numerical pain scale 0–10) [48];
(b) Tender points (counted by the same examiner at T1, T2, and T3 evaluation moments). The same physician conducted the tender point count for all patients at every evaluation instance. The 18 ACR-recommended points for FM [49], symmetrically positioned on the body, were assessed using the examiner’s thumb, applying pressure to the tested area until blanching of the nail bed occurred. A positive result was determined by the presence of pain upon pressure at the specified tender point. The locations of the painful points were then indicated on a diagram of the human body and tallied accordingly;
(c) Pain sensitivity to pressure assessed by pressure pain thresholds (PPTs).

Algometry was also administered to all participants using a digital algometer provided by Somedic (Norra Mellby, Sweden). This device applies pressure to the tissue, thereby stimulating the slow nociceptive peripheral fibers (C fibers). It does so at a consistent rate until the patient reaches their pain threshold, defined as the minimum pressure required for the individual to perceive the pressure as painful. The device comprises a round probe sensor connected to a pressure transducer and is equipped with a patient-operated switch. Patients were instructed to press the button on the switch as soon as they began to feel pain, enabling the device to record a numerical value (measured in kilopascals). The evaluator (the same examiner for all patients) applied pressure with the algometer until the patient operated the switch. At that point, the value was displayed on the LCD screen of the algometer and recorded by the evaluator. The algometer was set to B3, indicating the use of a 1-square-centimeter probe with a slope of 30 kilopascals per second. The algometer was applied to the evaluation area at a right angle and was stabilized between the investigator’s second and third fingers. Prior to use, the algometer was calibrated according to the manufacturer’s instructions. For cervical points, the patient lay prone, while for other points, they lay supine with arms relaxed on the bed and legs extended. Three measurement points were designated on each side of the body (left and right): lateral to the C5 vertebra, in the second metacarpal space, and 6 cm below the tibial tuberosity on the side of the calf, within the anterior tibial muscle. For each of the 6 points, three measurements were taken with a 10 s interval between them. This method adhered to the recommendations of the International Association for the Study of Pain and was consistently performed by the same evaluator for each assessment.

Functional impairment:

(a) Observed functional limitation expressed by assessing 8 of the most commonly used daily activities (crouching, lateral bending, climbing stairs, adducting and flexing the arms, lifting a 5 kg object, buttoning one’s shirt, walking straight, and hanging clothes). Each of these activities received a score between 1 and 4 (1 being without any difficulty, 4 being impossible to perform) given by two experienced investigators.
(b) Physical scale of the Fibromyalgia Impact Questionnaire (FIQ) [50], consisting of 10 items assessing daily function in a typical week. The higher the scores obtained, the more severe the impact of the disease.

Emotional consequences: To evaluate the anxiety and depression symptoms, we used the Hospital Anxiety and Depression Scale (HADS) [51]. It is a 14-item scale (7-item anxiety subscale and 7-item depression subscale) based on self-reported symptoms.

Disease impact: The disease impact was evaluated using the following:

(a) The patient’s perception of the disease, assessed through the total score of the Fibromyalgia Impact Questionnaire (FIQ);
(b) The medication intake, expressed as the number of medications the patient takes on a daily basis to control symptoms of FM. We evaluated the following aspects: the names of the medications, their usage duration, dosage, frequency, efficacy, and causes for interruption. For statistical analysis, we used the total number of tablets ingested by the patient on a daily basis.

### 2.6. Statistical Analysis

The Statistical Package for Social Sciences (SPSS) version 20 (IBM Corporation, Armonk, NY, USA) program was utilized to create and analyze the database. The validity of the premises of normality and homoscedasticity was verified first, given that the samples were larger than the limit of 30 subjects.

For the normality part, we used the Kolmogorov–Smirnov test, whose null hypothesis claims normality. It is rejected if the significance is smaller or equal to 0.05. Most of the considered variables satisfied this hypothesis, and for those who did not fulfill it, the standard error of the kurtosis was considered. It was observed that, despite not satisfying the normality criteria, none of the variables had limit values that would lead to their rejection from the parametric tests. Homoscedasticity was verified with the Levene test, whose null hypothesis claims the homogeneity of variables. It is rejected if the significance is smaller or equal to 0.05.

Descriptive analysis and comparisons of means and percentages were conducted to present the characteristics of the samples (both the interventional and control ones). Descriptive analysis was used to contextualize the sample within the sociodemographic and clinical parameters of interest. ANOVA for continuous and nominal variables was performed to analyze the potential differences of the variables between the two samples. Differential analysis was applied to each group to evaluate the evolution of the considered variables over time. For each group independently, mean comparisons were made (paired-samples T-Student test, repeated measures) to determine the significant differences of the parameters between the three evaluation moments (T1, T2, and T3). Variance analysis was performed using the independent-samples T-Student test and ANOVA (Huynh–Feldt correction was applied when the sphericity could not be assumed). The interpretation of the statistical significance [4] (*p*) for the difference in the considered variables between the treatment options, the moment of evaluation, or the correlation tendency was conducted according to the value of the significance threshold.

## 3. Results

### 3.1. Demographic Characteristics

The average age of the patients included in this study was 56 years, with insignificant differences between the studied groups (*p* < 0.01). Most of the patients had up to a secondary education, and less than 15% of them were working at the time of the intervention. In Table 1, the social and demographic data for each group are presented, with highly significant differences between groups being observed for education level and working status.

The onset of pain was reported, on average, 9 years prior to the diagnosis of fibromyalgia (FM). No statistically highly significant differences were observed between the groups in terms of comorbidities. We mention, in descending order, emotional disorders (anxiety or depression), tension headaches, and endocrine disorders. The mentioned elements are structured in Table 2.

### 3.2. Pain

a. The results of the average pain level rated by the patients on a numeric scale and the pain sensitivity to pressure for the monitored groups are shown in Table 3. For the group involved in kinesiotherapy, a highly significant decrease was registered between T1 and T2 (*p* = 0.000) and between T1 and follow-up (T3) (*p* = 0.000), with the difference between T2 and T3 not having any significance (*p* = 0.1). The group subjected to physical modalities (PM) also noted a highly significant difference between the T1 and T2 evaluations (*p* = 0.000), between T1 and T3 (*p* = 0.000), and between T2 and T3. Regarding the differences between the groups, significant disparities were observed at the T2 evaluation between the samples undergoing PM or KT (*p* = 0.000). These significant differences were maintained at the T3 stage between the groups engaged in kinesiotherapy and physical modalities (*p* = 0.000).
b. Also, the number of tender points registered significantly decreased between the initial evaluation and the last two ones for the group subjected to kinesiotherapy. For the sample involved in physical modalities, the number of tender points had the following evolution: from an initial 16.1 average, it significantly decreased (*p* = 0.001) to 14.2 at T2 and was kept at a similar level (14.5) until the follow-up evaluation (T3). Inter-group differences: The differences between the two groups were highly significant at all evaluations: T1 (*p* = 0.000), T2 (*p* = 0.000), and T3 (*p* = 0.000), with clearly higher tender point averages for the group implicated in physical modalities.

A strong correlation was found at a general level between the average pain rated by the patients on a numeric scale and the maximum (r = 0.807, *p* = 0.000) or minimum (r = 0.753, *p* = 0.000) level of pain evaluated on the same scale. This correlation tended to strengthen over time between the average and maximum levels of pain (r = 0.630 at T1, r = 0.821 at T2, r = 0.816 at T3) and between the average and minimum pain levels (r = 0.621 at T1, r = 0.733 at T2, r = 0.812 at T3).

**Figure 1:**
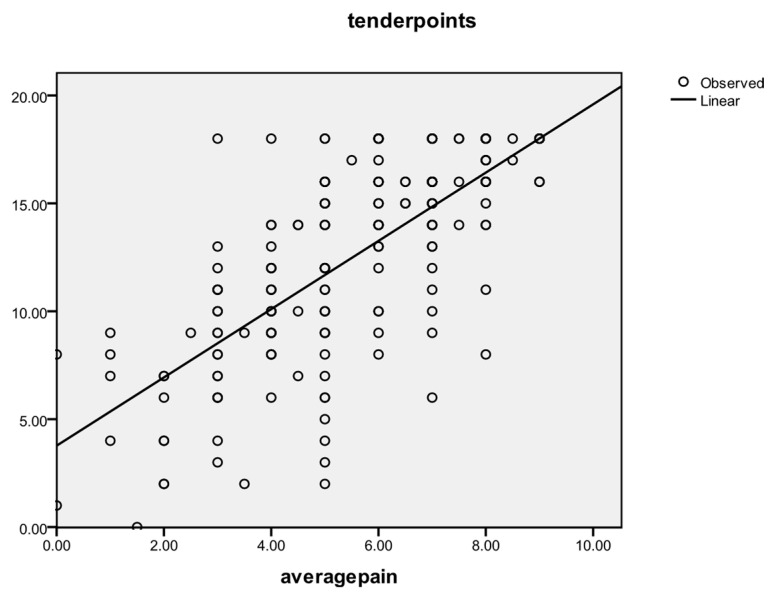
Correlation between average pain (VAS) level and tender points.

A general correlation tendency was observed for average pain and number of tender points (r = 0.708, *p* = 0.000), as shown in Figure 1. Initially, the value of the correlation coefficient was 0.487, but it grew over time to 0.626 after therapy and to 0.809 at follow-up.

Table 4 presents the evolution of the pressure pain thresholds measured with the digital algometer. Concerning the evolution of this variable over time, the most significant differences were noted for the group that underwent kinesiotherapy between the first and second evaluations for all monitored areas: the right cervical area (*p* = 0.001), the left cervical area (*p* = 0.014), the right hand (*p* = 0.036), the left hand (*p* = 0.009), the right tibial area (*p* = 0.000), and the left tibial area (*p* = 0.001). Between the T1 and T3 evaluations, significant differences could still be seen for the following areas: the right cervical (*p* = 0.003), the left hand (*p* = 0.01), and, especially, the tibial areas (the right one (*p* = 0.000) and the left one (*p* = 0.000)). There was a highly significant (*p* = 0.000) difference registered for all the monitored areas at all evaluation moments between the group that underwent kinetic therapy and the group subjected to physical modalities.

**Figure 2:**
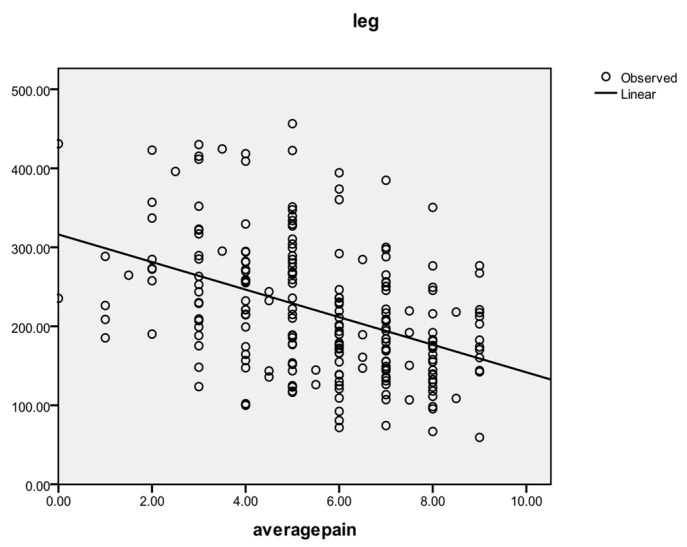
Correlation between average pain level and leg area threshold.

A reverse correlation was found between the average pain level and the pain tolerance measured by algometry for each of the three areas, as follows: cervical (r = −0.412, *p* = 0.000; r = −0.222 at T1, r = −0.518 at T2, r = −0.421 at T3), hand (r = −0.380, *p* = 0.000; r = −0.254 at T1, r = −0.465 at T2, r = −0.446 at T3), and tibial (r = −0.426, *p* = 0.000; r = −0.320 at T1, r = −0.507 at T2, r = −0.470 at T3). The general correlating tendency between average pain and leg threshold is shown in Figure 2.

### 3.3. Functional Impairment

Data regarding both the observed and perceived functional impairment have been collected in Table 5 for each of the evaluated time points.

a. For the observed functional impairment, the group involved in KT registered a significant decrease (*p* = 0.000) from the T1 moment (mean = 13.7, SD = 3.9) to the T2 moment (mean = 10.3, SD = 2.7), followed by an increase at T3 (mean = 12.4, SD = 3.5), which holds no statistical significance when compared to the first two evaluations. The group subjected to PM also registered a significant decrease (*p* = 0.000) between the scores at the T1 (mean = 15.4, SD = 2.6) and T2 (mean = 13, SD = 3.3) evaluations, a decrease (*p* = 0.000) that was still observed at T3 (mean = 13.8, SD = 3.1). The only significant difference (*p* = 0.001) between the two samples was registered at the second evaluation, with no significant differences observed at the T1 and T3 moments.
b. Where the perceived functional impairment is concerned, the initial score (mean = 12.1, SD = 5.3) for the KT group dropped significantly (*p* = 0.0) at T2 (mean = 9.8, SD = 5.3) and then increased at T3 (mean = 10.2, SD = 5.1), with the difference between the first and third evaluations not being significant (*p* = 0.137). For the group involved in PM, the initial score (T1) (mean = 12.7, SD = 4.6) decreased (*p* = 0.001) at T2 (mean = 8.6, SD = 7) and T3 (mean = 9.7, SD = 5.8), with statistical significance in the T3/T1 comparison.

### 3.4. Emotional Consequences

a. As shown in Table 6, with regards to the anxiety scale of the HADS inventory, significant differences were found between the first and second evaluations for both groups: for the group involved in kinesiotherapy, the initial score of 9.7 (SD = 3.1) dropped (*p* = 0.37) to 9 (SD = 3.3), while for the group that underwent physical modalities, a significant difference (*p* = 0.002) was observed due to the reduction in the score from 11.2 (SD = 3.5) to 8.1 (SD = 3.8). No important differences between the samples were registered at any evaluation moment.
b. The score computed from the depression scale of the HADS questionnaire displayed the following evolution over time: for the sample involved in kinesiotherapy, the score dropped (*p* = 0.05) from an initial 9.7 (SD = 2.8) to 8.3 after the interventional program, while for the group implicated in physical modalities, the reduction (*p* = −0.02) was from 11.2 (SD = 3.6) to 10 (SD = 3.8). Similar to the anxiety scale, no significant differences were observed between the two samples at any time (*p* > 0.05).

### 3.5. Disease Impact

The means and standard deviations obtained by computing the FIQ total score for both groups at all evaluation moments are presented in Table 7.

As it can be observed in Table 7, the scores from the group subjected to KT registered a significant decrease from T1 to T2 (*p* = 0.000) and T3 (*p* = 0.000). The group that underwent PM also registered a highly significant change between the initial (T1) and second scores (T2) (*p* = 0.000) and the initial (T1) and final ones (T3) (*p* = 0.000). Regarding the comparison of the two non-pharmacological treatments applied, it was evident that both had statistically highly significant effects on the functional level of the patients, both during the treatment period and at the T3 evaluation.

In terms of medication intake, the group involved in kinetic therapy displayed significant differences between T1 and T2 (*p* = 0.000), T2 and T3 (*p* = 0.005), and T1 and T3 (*p* = 0.000). Before starting the interventional program, the average use of medication for this sample was 3.9 tablets a day, while immediately after the 36-session program, the intake was 1.6, remaining at a low level (an average of 2 pills a day) even 4 months after the end of the intervention. For the sample subjected to physical modalities, the initial medication intake of 3.4 tablets a day remained at a similar level at T2 (3.3) and T3 (2.8). Comparing the two patient groups at the end of our study regarding the degree of reduction in medication use, it was evident that patients undergoing kinetic therapy exhibited an absolute reduction in the need for analgesic medication (*p* < 0.001).

## 4. Discussion

Patients with fibromyalgia often experience issues with balance, irrespective of the underlying reasons, which could include factors like aging, medication side effects, decreased muscle strength, or cognitive impairment. In a comprehensive survey involving 2596 individuals, 45% reported experiencing balance issues [52], primarily attributed to compromised mechanisms of postural control [53].

Supervised exercise programs have been shown to reduce the severity of fibromyalgia symptoms and effectively improve levels of pain, functional status, and quality of life. Typically, mixed exercise interventions encompass three primary components: aerobic and strength exercises [54,55], occasionally supplemented with relaxation exercises [56]. Additionally, Pilates programs are recognized for integrating physical, psychological, spiritual, and behavioral aspects, potentially offering significant advantages for individuals with fibromyalgia (FM) [57], who often present diverse physical and emotional challenges. This consideration influenced our decision to incorporate aerobic exercise and Pilates into the kinesiotherapy program. Altan et al. investigated the effects of Pilates training compared to home-based relaxation and stretching exercises on a group of FM women [42] and demonstrated better improvements in pain (rated on a visual analogue scale) and Fibromyalgia Impact Questionnaire results for the Pilates group, as have other authors [58]. Similar to other studies that investigated the effects of kinesiotherapy on pain [30,59,60,61], the results of the current study demonstrate that forms of kinesiotherapy, such as the combination of aerobic exercises and Pilates, influence pain both during treatment (*p* = 0.000) and three months after treatment (*p* = 0.000), regardless of whether we considered the pain reported by the patient, the objectification through tender points, or the use of algometry. The beneficial results obtained immediately after completing kinesiotherapy can be explained by the reduction in local inflammation and oxidative stress, leading to diminished stress responses [62]. Patients with fibromyalgia often display heightened levels of Substance P and decreased levels of serotonin, norepinephrine, and dopamine metabolites in their cerebrospinal fluid, indicating altered pain processing and reduced central regulation of sensory information [9]. Hence, the positive impacts on chronic pain might be clarified by diminishing pain through bolstering the body’s reaction to muscle microtrauma and facilitating the mechanisms of repair [63]. It is well known that women with FM have significantly lower perceived functional ability and demonstrate impaired physical performance compared to control patients of similar age [64]. Similar to other studies [65,66] regarding functional impairment and the consequences of the disease on the patient (FIQ), the combination of aerobic exercises with Pilates demonstrates, in the study group, the effectiveness of this non-pharmacological treatment both at the end of the 12-week training period and at the 3-month follow-up, despite the significance of this result diminishing over time (*p* = 0.07, T3/T2 evaluation; *p* = 0.000, T3/T1).

While there seems to be no single best treatment option, physical modalities in FM appear to improve disease consequences [63], but have been less studied than other forms of therapy regarding their benefits for FM patients. In this study, combining two forms of electrotherapy, TENS and low-level laser, along with thermotherapy, yielded notably positive outcomes in pain relief, as indicated by subjective assessment (VAS) (*p* = 0.000, T3/T1) and objective evaluation (tender point count) (*p* = 0.001, T2/T1). As anticipated, the pain alleviation was less pronounced during extended assessments of tender points (*p* = 0.02, T3/T1). The direct or indirect impacts of these three physical modalities stem from photochemical reactions, resulting in enhanced messenger RNA and adenosine triphosphate production, thus mitigating cell inflammation [41,67]. Nevertheless, these effects diminish over time following the discontinuation of the specified treatment modalities. Regarding functional impairment, both objective and perceived, the combined application of the three physical modalities demonstrated a highly significant improvement between the end of treatment (perceived functional impairment, *p* = 0.003, T2/T1) and the maintenance of statistically significant improvement (*p* = 0.03, T3/T1) at the evaluation three months after treatment cessation.

The published data refer to the effects of one form of physical modality or a combination of two forms (usually electrotherapy) [47,61,68]. The same authors demonstrated that electrotherapy in FM acts on pain, sleep, and quality of life [63]. Regarding the emotional consequences and the illness consequences, the combination of physical modalities has shown influence on anxiety-related elements (HAD anxiety, *p* = 0.007) even at the follow-up evaluation. We would consider that this effect is a result of the application of thermotherapy, which has a direct effect on muscle relaxation. A team of Spanish researchers investigated the effects of peloids as a form of thermotherapy on various categories of patients with rheumatological conditions. The conclusion of the 2021 study supports the beneficial effect on functional capacity and quality of life [69]. In the comparison between two forms of non-pharmacological therapy combinations—namely, aerobic exercises plus Pilates versus physical modalities (TENS plus LLT plus thermotherapy)—our study has shed light on several noteworthy findings. Concerning the impact on pain, kinesiotherapy (KT) appears to exhibit greater efficacy compared to physical modalities (PM) across all three evaluation types (subjective pain assessment, tender point count, and pain objectification through algometry). Statistically significant differences were observed at both the T2 evaluation (*p* = 0.000) and the three-month follow-up (*p* = 0.000). Furthermore, in terms of functionality—both objective and perceived—KT demonstrated superiority only at the conclusion of the treatment period (*p* = 0.001). However, there were no statistically significant disparities in the ability to influence emotional outcomes or the quality of life between kinesiotherapy and physical modalities. Notably, the assessment of pain and functional impairment proved to be less affected in intensity but over a longer duration (3 months) compared to the kinesiotherapy program. Moreover, our study did not identify existing research comparing the therapeutic methods utilized, suggesting these findings pose a challenge for future research in this field.

Limitations of this study:

Our study had some limitations. The main drawback was that we only included female patients from one university center and had a limited range of non-pharmacological treatments, both kinetic and physical. Additionally, the lack of research on non-pharmacological treatment, especially physical modalities, and the absence of studies comparing the effectiveness of these two treatment types equally posed a limitation for comparison in the Discussion section and presented a significant challenge.

## 5. Conclusions

In conclusion, the present study aimed to offer a more comprehensive therapeutic approach by combining different modalities of electrotherapy and thermotherapy, as opposed to the combination of kinesiotherapy targeting various movement types. Both therapies were found effective in alleviating pain and other associated symptoms of fibromyalgia, with the clear superiority of kinesiotherapy in terms of pain relief but with particular characteristics regarding the evolution of the studied parameters in patients from the physical modality group. However, further robust research is necessary to confirm the effectiveness of various non-pharmacological treatments for a condition whose underlying causes are still not fully understood. Given the unique characteristics of both the condition and the patients, it is worthwhile to consider tailoring treatments, especially if we have a range of effective non-pharmacological therapies available for other conditions.

## Figures and Tables

**Table 1:** Social and demographic data (KT = kinetic therapy group; PM = physical modality group; n = number of patients; SD = standard deviation).

Social and Demographic Data	KT (n = 34)	PM (n = 30)	*p* Value
Mean age (SD)	55.3 (7.2)	58.6 (6.9)	<0.01
No. studies	0%	0%	<0.001
Primary education	38.2%	33.3%	<0.01
Secondary education	41.2%	43.4%	<0.001
Higher education	20.6%	23.3%	<0.001
Working	14.7%	10%	>0.05
Unemployment	8.8%	6.6%	<0.05
Medically retired	61.8%	63.4%	<0.001

**Table 2:** Clinical diagnosis data (KT = kinetic therapy group; PM = physical modality group; n = number of patients; SD = standard deviation).

Clinical Data	KT (n = 34)	PM (n = 30)	*p* Value
Duration of illness: mean (SD)	3 (2)	7 (2.5)	<0.01
Duration of pain: mean (SD)	8 (7.3)	4 (8.6)	<0.01
Irritable bowel syndrome	23.5%	20.0%	<0.01
Chronic fatigue syndrome	20.6%	16.6%	<0.01
Tension headaches	52.9%	53.6%	<0.001
Endocrine disorders	41.2%	40.0%	<0.001
Anxiety or depression	52.9%	54.0%	<0.001
Other associated conditions	15.8%	12.3%	<0.01

**Table 3:** Average pain (Avg pain) rated on a numeric scale and tender points (tender *p*) (SD = standard deviation).

Avg Pain. Tender Pts	T1 Mean (SD)	T2 Mean (SD)	T3 Mean (SD)	T1/ T2 (*p*)	T3/ T2 (*p*)	T3/ T1 (*p*)
KT pain	6 (1.4)	3.4 (1.6)	4 (1.7)	0.000	0.1	0.000
PT pain	7.2 (1.2)	5.6 (1.5)	6.2 (1.7)	0.000	0.000	0.000
KT/PT (*p*)	0.000	0.000	0.000	-	-	-
KT tender *p*	12.9 (3.6)	6.9 (3.4)	9.6 (3.6)	0.000	0.000	0.000
PM tender *p*	16.1 (2.1)	14.2 (3.1)	14.5 (3)	0.001	0.3	0.02
KT tender *p*/PM tender *p* (*p*)	0.000	0.000	0.000	-	-	-

**Table 4:** Pressure pain thresholds (R = right; L = left; SD = standard deviation).

Group	Moment	CervicalR	CervicalL	HandR	HandL	TibialR	TibialL
KT Mean (SD)	T1	152 (38.7)	156 (35)	207.5 (48)	199.4 (43.1)	263.2 (59)	260.5 (70.7)
	T2	169.8 (45.4)	171.7 (40)	221.7 (43.2)	217.9 (54.3)	292.9 (79.3)	288.8 (75.6)
	T3	162.3 (35.5)	156.1 (28.5)	219.9 (41.3)	192.5 (33.8)	307.8 (63.3)	305.3 (66.4)
PM Mean (SD)	T1	111.3 (24.7)	104 (25)	144.5 (44.4)	145 (39.7)	174.5 (36.1)	164 (33.6)
	T2	114.4 (28.7)	110 (28.1)	139.6 (40.5)	141.2 (39.1)	204.8 (92)	155.6 (42.2)
	T3	114.1 (28.2)	108.3 (28.7)	141.5 (41.6)	139.4 (38.1)	173.3 (34.5)	157.9 (34.2)

**Table 5:** Functional impairment (observed and perceived values) (SD = standard deviation).

	T1	T2	T3	T1/T2 (*p*)	T3/T2 (*p*)	T3/T1 (*p*)
Observed FI (KT)	13.7(3.9)	10.3(2.7)	12.4(3.5)	0.000	0.007	0.153
Observed FI (PM)	15.4(2.6)	13.0(3.3)	13.8(3.1)	0.003	0.337	0.034
Observed KT/FM (*p*)	0.047	0.001	0.097	-	-	-
Perceived FI (KT)	12.1(5.3)	9.8(5.3)	10.2(5.1)	0.078	0.752	0.137
Perceived FI (PM)	12.7(4.6)	8.6(7.0)	9.7(5.8)	0.010	0.510	0.030
Perceived KT/FM (*p*)	0.633	0.439	0.715	-	-	-

**Table 6:** HADs inventory (anxiety and depression scale values) (SD = standard deviation).

	T1	T2	T3	T1/T2 (*p*)	T3/T2 (*p*)	T3/T1 (*p*)
HAD anxiety (KT)	9.7 (3.1)	9.0 (3.3)	9.5 (3.4)	0.371	0.540	0.801
HAD anxiety (PM)	11.2 (3.5)	8.1 (3.8)	8.6 (3.7)	0.002	0.608	0.007
HAD anxiety Kt/PM (*p*)	0.074	0.314	0.315	-	-	-
HAD depression (KT)	9.7 (2.8)	8.3 (3.1)	9.5 (2.8)	0.055	0.099	0.769
HAD depression (PM)	11.2 (3.6)	10.0 (3.8)	10.0 (3.8)	0.214	NaN	0.214
HAD depression Kt/PM (*p*)	0.066	0.053	0.548	-	-	-

**Table 7:** Total FIQ scores (SD = standard deviation).

Group	FIQ Total T1	FIQ Total T2	FIQ Total T3	T1 vs. T2 (*p*)	T1 vs. T3 (*p*)
KT mean (SD)	59.3 (18.9)	43.4 (18.6)	45.1 (17.9)	0.000	0.000
PM mean (SD)	72.5 (10.5)	51.0 (18.1)	55.6 (17.3)	0.000	0.000
KT/PM (*p*)	0.001	0.104	0.020	-	-

## Data Availability

Data are contained within the article.
